# Acceptability of Hormonal Contraceptives as a Smoking Cessation Aid for Women of Reproductive Age: A Web-Based Cross-Sectional Survey

**DOI:** 10.1089/whr.2023.0130

**Published:** 2024-02-22

**Authors:** Samantha J. Werts-Pelter, Briana M. Choi, Stephanie Mallahan, Nicole Person-Rennell, Alicia Allen

**Affiliations:** ^1^ Department of Health Promotion Sciences, Mel and Enid Zuckerman College of Public Health University of Arizona, Tucson, Arizona, USA.; ^2^ Department of Pharmacy Practice and Science, R. Ken Coit College of Pharmacy, University of Arizona, Tucson, AZ.; ^3^ Clinical Translational Sciences, College of Medicine, University of Arizona, Tucson, Arizona, USA.; ^4^ Department of Family and Community Medicine, College of Medicine, University of Arizona, Tucson, Arizona, USA.

**Keywords:** tobacco, addiction, women's health, hormones, health disparities, cancer

## Abstract

**Introduction:**

Cigarette smoking is the most common cause of preventable cancers and other premature morbidity and mortality. Modifying hormonal patterns using hormonal contraceptives (HCs) may lead to improved smoking cessation outcomes in women, though the acceptability of this is unknown. Therefore, we explored the willingness of reproductive-age women who smoke to use HC for cessation.

**Methods:**

A cross-sectional online survey was conducted with a convenience sample of reproductive-age women living in the United States who self-reported smoking combustible cigarettes. Questions covered smoking history, previous HC use, and willingness to use various HC methods (*i.e*., injectable, oral, patch, vaginal insert) for cessation. Chi-squared tests and logistic regression were conducted using StataBE 17.1.

**Results:**

Of 358 eligible respondents, *n* = 312 (86.9%) reported previous HC use. Average age of those with HC use history was 32.1 ± 6.1 years compared with 27.8 ± 6.7 years for those without history of HC use (*p* = 0.001). Of respondents who reported previous HC use, 75.6% reported willingness to use HCs, compared with 60.9% of those without a history of HC use. Overall, willingness to use various types of HC ranged from 22.6% for the vaginal insert to 59.2% willing to use an oral contraceptive.

**Discussion:**

These observations indicate that most women who smoke cigarettes are willing to use HC for a smoking cessation aid, especially if they have a history of HC use and with an oral form of HC. To improve the rate of smoking cessation for women of reproductive age, future interventions should explore how to incorporate HC for cessation.

## Introduction

An estimated 20 million women smoke combustible cigarettes in the United States.^1^ Though 75% of women who smoke report wanting to quit, fewer than 3% are successful each year.^2,3^ Compared with men, women are more likely to smoke for non-nicotine reinforcing factors such as management of weight, mood, and/or stress.^4–8^ Consequently, most smoking cessation pharmacotherapies are less efficacious in women, including nicotine replacement therapy (NRT), which is the most accessible form.^2,8,9^

Ultimately, women are more likely to relapse compared with men.^8,10^ The lack of effective smoking cessation interventions for women is a public health concern, given women who quit smoking combustible cigarettes by age 40 can reduce their smoking-related mortality up to 90%.^11^

Ovarian hormones (*i.e*., progesterone, estrogen) vary throughout the menstrual cycle. Fluctuations in these ovarian hormones affect smoking-related symptomatology (*e.g*., craving, withdrawal) and, perhaps, susceptibility to smoking relapse.^12–14^ Preclinical evidence indicates that high estrogen increases nicotine demand and use.^15,16^ In contrast, high progesterone reduces nicotine administration.^14,17^ While clinical observations are more mixed, these relationships have been demonstrated in naturally cycling women.^12,13,18–21^

Hormonal contraceptives (HCs) also influence combustible cigarette smoking by altering affect,^22–25^ anxiety/stress,^26–30^ withdrawal,^26,31^ craving,^24,32^ response to nicotine,^26,33^ and nicotine metabolism.^26,34,35^ Alteration of these known smoking relapse risk factors may lead to differences in smoking relapse risk. Indeed, our preliminary work indicates that 39% of those using HCs were abstinent 6 months after an assigned quit date in recently completed smoking cessation clinical trials as compared with 25% women not using HCs and 33% of men.^24^

While these data are still emerging, one theory is that because the use of HCs blocks ovulation—resulting in the reduction of the ovarian hormonal fluctuations that occur with the natural menstrual cycle and reduction in estrogen levels^36^—there may be a decreased smoking relapse risk.^37^ Despite these promising observations, it is important to note that ethinyl estradiol (the synthetic form of estrogen commonly used in combination oral contraceptives) is contraindicated in women who smoke or those over the age of 35 due to an increase in cardiovascular issues.^38,39^

In contrast, forms of HC that contain only progestin (a synthetic form of progesterone) are considered safe for use in women who smoke and/or are older.^40,41^ Together, these observations indicate that there is promise for HCs to aid in smoking cessation efforts in women; however, the risks also need to be carefully considered.

Delivery of exogenous natural progesterone has been evaluated for smoking cessation with randomized clinical trials indicating that it decreases risk of smoking relapse in pregnant^1,2^ and non-pregnant^42^ women of reproductive age with success. However, to date, purposeful reduction of ovarian hormonal fluctuation and/or estrogen to reduce smoking has not yet been examined.

In addition, HCs are used off-label to treat a variety of health issues (*e.g*., acne, endometriosis), though they have not yet been used for smoking cessation.^37,43^ Therefore, we sought to assess the willingness of reproductive-age women who smoke to use various types of HCs, including synthetic exogenous hormones (*i.e*., ethinyl estradiol, progestin) and administrations (*i.e*., injectable, oral, patch, vaginal insert), for smoking cessation.

## Methods

### Study design and sample

We recruited a convenience sample via an anonymous, cross-sectional survey conducted on an online survey panel site (Prolific) between December 2021 and January 2022. Individuals with profiles on the survey panel site who identified as women living in the United States were invited to complete the survey through REDCap,^44^ hosted at the University of Arizona. Respondents were compensated ∼$5 by the survey panel site after completion was verified by the research team.

The survey on average took 20 minutes to complete and included four attention check questions to increase reliability of the data. Eligibility criteria for this study included: people who reported they were female at birth, 18–40 years of age, and self-reported currently using combustible cigarettes. Participants were excluded if they identified as gender queer or transgender and if they did not answer correctly on one or more of the attention check questions. This study was approved by the University of Arizona Institutional Review Board (IRB: 2006754153).

### Measures

The survey contained researcher-created questions regarding demographic information (*i.e*., age, gender, race, ethnicity, education), previous and current HC use, willingness to use various forms and types of HC for cessation, and qualitative open-ended questions regarding why or why not participants want to utilize different forms and types of HC as a smoking cessation aid (Supplemental File S1). For previous HC use, participants were asked “have you ever used any form of hormonal birth control” with answer options of “yes,” “no,” “unsure,” and “prefer not to answer.”

Those who answered “unsure,” and “prefer not to answer” for this question were excluded from analyses. For those who indicated they have used HC, participants were then prompted with several of the common forms of hormonal birth control and asked if they were currently using it, had used it within the past 1–3 months, or had used it over 3 months ago.

Several HC administration modes and types were assessed, as seen in Table 2. We selected these options based on currently available forms of HC that are less invasive forms of contraceptive as compared with the intrauterine device or implant.^37^ In addition, because exogenous administration of ethinyl estradiol is contraindicated in women over the age of 35 and those who smoke while progestin is safe,^45^ we also wanted to assess the perceptions of safety for the differing hormones. For each HC type participants indicated they were unwilling to use, an additional short-answer, open-ended question was prompted to ask why they were unwilling to use that type.

Willingness to use different methods of HC was assessed via a 5-point Likert scale ranging from “not at all willing” to “very willing.” For analysis, responses of “not at all willing” and “somewhat not willing” were combined as a category of “not willing.” “Neutral” was categorized as “neutral.” “Somewhat willing” and “very willing” were combined as “willing.”

### Data analyses

Analyses included frequencies and percentages for categorical data and mean and standard deviation for continuous data. For the open-ended questions regarding willingness to use HC as a smoking cessation aid, responses were analyzed for common answers by one reviewer (B.C.) using a content analysis approach.

The coder created a list of the responses based on commonalities and then quantified each response type. As these were short responses, no additional thematic content was assessed beyond frequency of response type. Differences between those with and without previous HC use were assessed using a chi-squared test and *t*-test.

Chi-squared tests were used to analyze the relationship between previous history of HC use and willingness to use HC for smoking cessation. Logistic regression was conducted to determine any differences when stratified by age, race/ethnicity, and motivation to quit (data not shown).

Statistical analyses were conducted using StataBE 17.1 (StataCorp LLC, College Station, TX). For all analyses, adjusted logistic regression analyses were conducted to compare differences by descriptive variables. Unadjusted and adjusted odds ratios and 95% confidence intervals were calculated. Missing data were excluded from analyses, and the alpha level was set at 0.05.

## Results

### Participants

Of the 509 survey respondents, 358 participants met eligibility criteria and were included in analysis. Reasons for exclusion included lack of current combustible cigarette use (*n* = 150, 29.5%), failure of attention checks (*n* = 2, 0.4%), and/or missing data (*n* = 31, 6.1%). Most participants identified as white (90.8%), non-Hispanic (88.3%), and had some college or technical school experience (42.7%) (Table 1).

**Table 1.** Description of Survey Respondents by History of Hormonal Contraceptive Use (*n* = 358)

Characteristics—***n*** (%)	Total ***n*** = 358 (100)	History of HC use ***n*** = 312 (86.9)	No history of HC use ***n*** = 46 (12.8)	***p*** ^a^
Cigarette use frequency,^*^ in the past 7 days				0.03
Every day	254 (71.0)	223 (71.5)	31 (67.4)	
Some days	81 (22.6)	73 (23.4)	8 (17.4)	
Not at all	23 (6.4)	16 (5.1)	7 (15.2)	
Length of cigarette use				0.21
Less than 1 year	27 (10.6)	21 (9.4)	6 (19.4)	
1–3 years	36 (14.2)	30 (13.5)	6 (19.4)	
More than 3 years	190 (74.8)	171 (76.7)	19 (61.3)	
Currently uses electronic cigarettes				0.60
Yes	120 (33.5)	103 (33.0)	17 (37.0)	
No	238 (66.5)	209 (67.0)	29 (63.0)	
Electronic cigarette use frequency, in the past 7 days				0.56
Every day	51 (42.5)	44 (42.7)	7 (41.2)	
Some days	63 (52.5)	53 (51.5)	10 (58.8)	
Not at all	6 (5.0)	6 (5.8)	0 (0)	
Past quit attempts				0.36
3+	110 (30.6)	99 (31.7)	11 (23.9)	
1–2	173 (48.3)	149 (47.8)	24 (52.2)	
0	69 (19.3)	60 (19.2)	9 (19.6)	
Interest in quitting				0.01
Not interested	61 (17.0)	46 (14.7)	15 (32.6)	
Interested	247 (69.0)	218 (69.9)	29 (63.0)	
Not sure	50 (14.0)	48 (15.4)	2 (4.4)	
Type of HC used^b^, current or previous				
Birth control pills	282 (78.6)	282 (90.4)	-	
Intrauterine device	80 (22.3)	80 (25.6)	-	
Injectable (*i.e*., Depo Provera)	73 (20.3)	73 (23.4)	-	
Transdermal patch	39 (10.9)	39 (12.5)	-	
Implant (*i.e*., Nexplanon)	39 (10.9)	39 (12.5)	-	
Contraceptive ring	40 (11.1)	40 (12.8)	-	
Emergency contraceptive	143 (39.8)	143 (45.8)	-	
Race				
Black	24 (6.7)	19 (6.1)	5 (10.9)	0.23
Asian, Native Hawaiian, or Pacific Islander	8 (2.2)	4 (1.3)	4 (8.7)	0.001
American Indian or Alaskan Native	11 (3.1)	11 (3.5)	0 (0)	0.20
White	325 (90.8)	285 (91.4)	40 (87.0)	0.34
Other	6 (1.67)	4 (1.28)	2 (4.4)	0.13
Ethnicity				
Hispanic	32 (8.9)	24 (7.7)	8 (17.4)	0.03
Non-Hispanic	317 (88.3)	281 (90.1)	36 (78.3)	0.02
Other	7 (2.0)	6 (1.9)	1 (2.2)	0.91
Age—mean (standard deviation)	30.8 (6.3)	31.2 (6.1)	27.8 (6.7)	0.001
Education				0.34
Less than high school or high school diploma or GED	63 (17.7)	58 (18.7)	5 (11.1)	
Some college or tech school	152 (42.7)	133 (42.8)	19 (42.2)	
College graduate	119 (33.4)	99 (31.8)	20 (44.4)	
Graduate school degree	22 (6.2)	21 (6.8)	1 (2.2)	

^*^ All respondents endorsed use of combustible cigarettes within the past 30 days. *n* = number of participants.

^a^ Determined using chi-squared test for categorical variables and *t*-test for continuous variables.

^b^ Categories are not mutually exclusive, and participants could report use of more than one type.

HC, hormonal contraceptive.

Mean participant age was 30.8 ± 6.3 years. Of the 358 participants, 312 (86.9%) reported previous or current use of HCs and 46 (12.8%) reported no previous use. Individuals with previous HC use were significantly older (31.2 ± 6.1 years) than those without a history of use (27.8 ± 6.7 years; *p* = 0.001). A significantly larger proportion of those with previous HC use identified as non-Hispanic (90.1%) than those without previous HC use (78.3%; *p* = 0.02).

Cigarette use frequency and interest in quitting also differed significantly by group. A greater percentage of those with previous HC use reported smoking every day or some days in the past week (94.9%) than those without previous HC use (84.8%; *p* = 0.03). Though the number of previous quit attempts did not differ by group, those without previous HC use were more likely to report that they are not interested in quitting at this time (32.6%) compared with those with previous HC use (14.7%; *p* = 0.01).

For those who reported a previous quit attempt, 60 (16.8%) reported attempting to quit “cold turkey,” without any additional resources or tools (Supplementary Table S1). Most who used a cessation aid reported using NRT, in the form of gum (28.2%) and/or patches (26.0%). Less than 10% reported using medications, state quitline, and/or counseling for cessation.

A total of 120 participants reported currently using electronic cigarettes, with 15 participants (4.2%) reporting vaping as an active cessation method for combustible cigarettes. Most (69.0%) of respondents indicated that they were motivated to quit tobacco use now (18.7%), in the next month (13.4%), 1 to 3 months from now (18.4%), or more than 3 months from now (18.4%), compared with 31.0% who were not planning to quit or were unsure.

When asked about their willingness to use various cessation aids in the future, most indicated that they would be willing to use NRT patches (46.1%), NRT gum (44.4%), or NRT lozenges (41.9%). Respondents who endorsed previous HC use were significantly more likely to indicate their willingness to use these aids than those who had not previously used HCs. Respondents were least likely to endorse willingness to use a state quitline (21.2%).

### Willingness to use HC for cessation

When asked about willingness to use HCs for cessation, our results show that the majority of our sample is willing to use at least one form of HC for smoking cessation (73.7%). Respondents who reported a history of HC use were more willing to use at least one type of HC for smoking cessation (75.6%) than respondents who reported no history of HC use (60.9%) (Table 2).

**Table 2.** Willingness to Use Hormonal Contraceptives for Smoking Cessation by History of Hormonal Contraceptive Use (*n* = 358)

Types of HC—***n*** (%)	Total—***n*** = 358 (100)	History of HC use ***n*** = 312 (86.9)	No history of HC use ***n*** = 46 (12.8)	***χ^2^ p***-value
Injectable, lasting 3 months				7.66 *p-* = 0.05
Willing	166 (46.4)	152 (48.7)	14 (30.4)	
Neutral	44 (12.3)	34 (10.9)	10 (21.7)	
Not willing	146 (40.8)	124 (39.7)	22 (47.8)	
Injectable, lasting 1 month				4.26 *p* = 0.24
Willing	140 (39.1)	128 (41.0)	12 (26.1)	
Neutral	60 (16.8)	50 (16.0)	10 (21.7)	
Not willing	156 (43.6)	132 (42.3)	24 (52.2)	
Injectable, lasting 1 week				1.41 *p* = 0.70
Willing	141 (39.4)	126 (40.4)	15 (32.6)	
Neutral	51 (14.3)	44 (14.1)	7 (15.2)	
Not willing	164 (45.8)	140 (44.9)	24 (52.2)	
Self-injectable				2.14 *p* = 0.54
Willing	131 (36.6)	117 (37.5)	14 (30.4)	
Neutral	44 (12.3)	36 (11.5)	8 (17.4)	
Not willing	180 (50.3)	156 (50.0)	24 (52.2)	
Injectable, administered by health care provider				5.67 *p* = 0.13
Willing	167 (46.7)	152 (48.7)	15 (32.6)	
Neutral	60 (16.8)	48 (15.4)	12 (26.1)	
Not willing	129 (36.0)	110 (35.3)	19 (41.3)	
Oral				6.42 *p* = 0.09
Willing	212 (59.2)	192 (61.5)	20 (43.5)	
Neutral	48 (13.4)	41 (13.1)	7 (15.2)	
Not willing	97 (27.1)	78 (25.0)	19 (41.3)	
Transdermal patch				3.58 *p* = 0.31
Willing	181 (50.6)	163 (52.2)	18 (39.1)	
Neutral	59 (16.5)	48 (15.4)	11 (23.9)	
Not willing	117 (32.7)	100 (32.1)	17 (37.0)	
Vaginal insert				3.34 *p* = 0.34
Willing	81 (22.6)	75 (24.0)	6 (13.0)	
Neutral	56 (15.6)	48 (15.4)	8 (17.4)	
Not willing	218 (60.9)	186 (59.6)	32 (69.6)	
Estrogen-containing				9.95 *p* = 0.02
Willing	147 (41.1)	137 (43.9)	10 (21.7)	
Neutral	89 (24.9)	76 (24.4)	13 (28.3)	
Not willing	119 (33.2)	96 (30.8)	23 (50.0)	
Progesterone-containing				12.08 *p* = 0.007
Willing	147 (41.1)	137 (43.9)	10 (21.7)	
Neutral	88 (24.6)	77 (24.7)	11 (23.9)	
Not willing	120 (33.5)	95 (30.5)	25 (54.4)	
Estrogen and progesterone containing				12.53 *p* = 0.006
Willing	143 (39.9)	134 (43.0)	9 (19.6)	
Neutral	91 (25.4)	79 (25.3)	12 (26.1)	
Not willing	121 (33.8)	96 (30.8)	25 (54.4)	

*n* = number of participants.

Regardless of history of HC use, overall respondents were most willing to utilize oral HCs for smoking cessation among contraception options (59.2%). In contrast, participants were least willing to use injectable HC, especially if they were self-injectable HCs (36.6%), and vaginally-inserted HCs (22.6%). Relating to the specific type of hormones used, those with a history of HC use were significantly more likely than those without previous history of HC use to be willing to use estrogen (43.9% vs. 21.7%, *p* = 0.02), progesterone (43.9% vs. 21.7%, *p* = 0.007), and a combination estrogen-progesterone (43.0% vs. 19.6%, *p* = 0.006) contraceptives.

The overall percentage of those willing to use cessation aids in the future was higher for the HC aids surveyed than the non-HC cessation aids, regardless of previous hormone use.

### Concerns for using HC for cessation

All participants who indicated they were unwilling to use a type of HC (Table 2) were queried about what factored into their decision. Our content analysis found that for all HC types except for oral contraceptives, the primary issue relayed was that participants were concerned with the mode of delivery. The largest mode of delivery concern was for people unwilling to use a self-administered injection HC (*n* = 113, 92.6%), followed by vaginally inserted HC (*n* = 98, 67.6%).

Just under half of those unwilling to use transdermal HC were concerned with the mode of delivery (*n* = 32, 40.5%). Participants unwilling to use oral HC were largely concerned with the hormones included in the medication (*n* = 25, 39.0%) and potential side effects, such as weight gain (*n* = 19, 29.7%). Additional concerns mentioned for all HC types included concerns about contraindication with current prescriptions or use of HC (*n* = 10), lack of information/knowledge about HCs (*n* = 9), concerns about the cost (*n* = 3), and lack of trust in the provider (*n* = 1).

## Discussion

The goal of this web-based, cross-sectional anonymous survey was to identify the willingness of reproductive-age self-identified women who currently smoke combustible cigarettes to utilize HCs for smoking cessation. We also wanted to determine if willingness differs by history of HC use. Our results show that the majority of our sample would be willing to use at least one form of HC for smoking cessation (73.7%). In addition, most of our sample had previously used HC (86.9%) and this experience was associated with a greater likelihood of willingness to use HC for smoking cessation.

Consistent with our findings, previous research has identified that individuals who smoke have a high prevalence of current or previous HC use.^32,46,47^ When asked about different types of administration, respondents were most willing to utilize the oral form and least willing to utilize a more invasive form of HC, such as an injectable or vaginally-inserted form. Data from the 2015 to 2017 National Survey of Family Growth indicate that, regardless of smoking history, oral contraceptives were the most commonly used form of contraception for people of reproductive age.^48^

As with our findings, a 2018 cross-sectional study identified that oral delivery was the most commonly used HC among individuals who smoke.^32^ Though willingness to use an orally delivered HC for smoking cessation aid may be related to familiarity with oral contraceptives, qualitative findings from the current study indicate that there are also concerns with other delivery modes being too invasive, which may impact their acceptability.

One of the identified barriers to the willingness to use HC for smoking cessation included the anticipation of weight gain. There is a well-documented evidence base that shows the relationship between weight concerns and smoking in women. Women commonly report that they smoke to help manage their weight.^49^ Compared with men, women are also significantly more likely to perceive weight gain as a negative outcome of smoking cessation and concerns about weight gain decrease motivation for smoking cessation.^50^

In addition, a systematic review completed in 2021 found 16 studies qualitatively identifying anxiety about weight gain as a primary reason for rejecting the use of HCs.^51^ For these reasons, using an HC specifically to aid in smoking cessation may compound these individual fears. Regardless of perception, there is little evidence showing that short-term (*e.g*., less than 3 months) use of HC is associated with significant weight gain,^52^ especially for progestin-only contraceptives such as injectable depot medroxyprogesterone acetate. HCs administered for smoking cessation intervention would also likely be limited in duration as a short-term modulator of ovarian hormone levels.

However, the impact of the use of HCs on weight gain has yet to be examined within the context of smoking cessation. Future research should identify the impact of HC use during cessation, as well as how the risk-benefit balance between potential weight gain and successful smoking cessation and, if applicable, effective messaging for both health care providers and women to inform shared decision making.

Currently, the evidence indicates that progesterone protects against drug-taking behaviors whereas estrogen facilitates these behaviors^14^; however, the literature is somewhat mixed in the clinical research on the role of these endogenous hormones in cigarette smoking.^12,13^ While the literature on the role of HCs in cigarette smoking and cessation is beginning to develop, it does indicate that use of HC may influence withdrawal, craving, affect, nicotine dependence, nicotine metabolism, physiological response to nicotine, and cessation.^46^

However, the use of ethinyl estradiol should be cautiously considered given the contraindications for use in women over the age of 35 and those who smoke,^45^ as well as preclinical research indicating that ethinyl estradiol may have adverse impacts on nicotine consumption.^15^ Overall, this study has demonstrated that use of HCs for smoking cessation is largely acceptable for women of reproductive age. When we asked respondents about their willingness to use the different types of hormones (estrogen- and/or progesterone-containing), there was no difference in willingness by type of hormones.

This may signal a general lack of awareness regarding the increased risk associated with the use of ethinyl estradiol as opposed to progestin. Thus, this may be an important point of education in future research and clinical efforts should HCs be examined and, ultimately, used for smoking cessation. The use of other types of exogenous hormones such as natural progesterone for treating menopausal symptoms or preventing preterm birth may also be considered for smoking cessation.^37^ Intervention implementation considerations should be made regarding delivery mode and education on anticipated adverse effects (and lack thereof).

Future work should investigate the ideal initiation (*e.g*., before, during, or after quit date), length of treatment to support cessation efforts in women of reproductive age, and reducing barriers (*e.g*., weight gain fears).

While this study explored a novel area of cessation research, it had some limitations to consider. First, this convenience sample was selected from respondents to an online survey panel site and may contain selection bias. Evidence shows that online panel respondents tend to be higher educated and have a higher income than the general population.^53^

Our sample was predominately non-Hispanic white with at least some college or technical school experience, compared with the general population of women who smoke in the United States, which is more prevalent in those of other race/ethnicity groups and those with less than a high school diploma.^3^ We also did not include a measure of nicotine dependence and, thus, it is unknown how dependent this sample may be.

Overall, these limitations reduce the generalizability of our observations. Second, self-reported data are prone to error, especially with regard to combustible cigarette use without biochemical confirmation, and there is a risk of straight-lining on online surveys,^54^ where participants answer quickly or with false answers to finish the survey. While this may be of lesser concern given our use of attention check questions and subsequent restriction of those who did not pass these questions, it is possible that this error was present in the final dataset.

In conclusion, these findings indicated that the majority of reproductive age women who smoke combustible cigarettes are willing to use HCs for smoking cessation. This is especially true for orally delivered HCs and among those with a history of HC use. To increase successful cessation outcomes for women of reproductive age, future work should examine how HCs may be used in conjunction with or independent of existing evidence-based approaches for smoking cessation.

## Supplementary Material

Supplemental data

Supplemental data

## Abbreviations Used

HC: hormonal contraceptive
IRB: Institutional Review Board
NRT: nicotine replacement therapy
