# Supplementary material for: Acceptability of Hormonal Contraceptives as a Smoking Cessation Aid for Women of Reproductive Age: A Web-Based Cross-Sectional Survey
Source: Womens Health Rep (New Rochelle). 2024 Feb 22;5(1):161–9. doi: 10.1089/whr.2023.0130 (PMC10898237; doi:10.1089/whr.2023.0130)
Supplement: Supplemental data [file Suppl_FileS1.docx]

**Supplementary File 1:** Survey Questions

**Tobacco Use**

Do you currently use cigarettes?

a. Yes

i. Thinking about the past seven days, how often did you smoke cigarettes?

1. Every day

How long have you been smoking at this rate?

- Less than 6 months
- Between 6 months and a year
- Between 1-3 years
- Over 3 years
- I’m not sure
- Prefer not to answer

1. Some Days
2. Not at all
3. Prefer not to answer

b. No

c. Prefer not to answer

Do you currently use e-cigarettes, JUUL, or vape?

a. Yes

b. No

c. Prefer not to answer

**Hormone Use**

Have you ever used any form of hormonal birth control?

1. Yes
2. No
3. Unsure
4. Prefer not to answer

What type of hormonal birth control have you used?

1. Birth control pills
2. Depo-Provera, an injectable (or shot) given once every three months
3. Contraceptive patch
4. Contraceptive ring or Nuvaring
5. Intrauterine device, IUD (also known as Mirena, Copper-T, or Paragard)
6. hormonal implant (also known as Norplant or Implanon)
7. emergency contraception (also known as Plan B or Morning After Pill)
8. Another type of hormonal contraception

[For each type of hormonal birth control that participants indicated they had used] Are you currently using this HC?

- 1. Currently using
  2. Not currently using, but used less than one month ago
  3. Not currently using, but used 1-3 months ago
  4. Not currently using, but used more than 3 months ago
  5. Prefer not to answer

**Quitting Interests**

Do you want to quit using tobacco?

1. Yes, I am trying to quit tobacco right now
2. Yes, I am going to try to quit tobacco within the next 30 days
3. Yes, I am going to try to quit tobacco in the next 1-3 months
4. Yes, I am going to try to quit tobacco in 3 or more months from now
5. No, I don’t plan on quitting tobacco
6. I am not sure
7. Prefer not to answer

Have you ever tried to quit using tobacco before?

1. Yes, once or twice
2. Yes, many times
3. No, I’ve never tried
4. I am not sure
5. Prefer not to answer

**Receptivity to Using Hormones to Quit**

The latest research shows that some female hormones (like progesterone and estrogen) influence how difficult it is for women to quit. We are interested in changing women’s hormones temporarily so that they may have an easier time quitting smoking. Assuming changing your hormones temporarily would be a safe thing to do, how willing would you be to try any of the following hormones as cessation methods?

1. An injectable hormone that lasts for three months
2. An injectable hormone that lasts for one month
3. An injectable hormone that lasts for one week
4. An injectable hormone that you inject into yourself
5. An injectable hormone that a health professional injects you with
6. An oral pill that you would take daily
7. A patch that would be placed on your skin for one month
8. A vaginal insert that would be inserted for one month
9. A hormone containing estrogen
10. A hormone containing progesterone
11. A hormone containing both estrogen and progesterone

[For each type of hormone that participants answered that they would be ‘not at all willing’ or ‘somewhat unwilling’ to use] Why are you not at all willing or somewhat unwilling to use ____? [open-ended responses]

**Demographics**

How old are you? _____

What gender do you identify with?

1. Male
2. Female
3. Transgender Male/Trans Man/FTM
4. Transgender Female/Trans Female/MTF
5. Gender Queer/Gender non-conforming
6. Prefer to self-describe as: ______
7. Prefer not to answer

How would you describe your race? (Select all that apply)

1. Black or African American
2. American Indian or Alaskan Native
3. Asian, Native Hawaiian or Pacific Islander
4. White or Caucasian
5. Other
6. Prefer not to answer

How would you describe your ethnicity?  (Check all that apply)

1. Hispanic
2. Not Hispanic
3. Other
4. Prefer not to answer

What is your highest level of education?

1. Less than high school graduate
2. High school diploma or GED
3. Some college or tech school
4. College graduate
5. Graduate degree (Masters or Doctorate)
6. Prefer not to answer
