# Supplementary material for: Acceptability of Hormonal Contraceptives as a Smoking Cessation Aid for Women of Reproductive Age: A Web-Based Cross-Sectional Survey
Source: Womens Health Rep (New Rochelle). 2024 Feb 22;5(1):161–9. doi: 10.1089/whr.2023.0130 (PMC10898237; doi:10.1089/whr.2023.0130)
Supplement: Supplemental data [file Suppl_TableS1.docx]

**Supplementary Table 1.** Respondent reports of cessation aids utilized previously and percentage of those willing to use resources for smoking cessation in the future by history of HC use (n=358).

| **Characteristics – n (%)** | **Total**  **n=358** | **History of HC Use**  **n=312 (86.9)** | **No History of HC Use  n=46 (12.8)** | **X^2^**  **p-value** |
| --- | --- | --- | --- | --- |
| **Previously Used Cessation Methods^a^** |  |  |  |  |
| NRT Patches | 93 (26.0) | 84 (26.9) | 9 (19.6) | 1.13  p=0.29 |
| NRT Gum | 101 (28.2) | 92 (29.5) | 9 (19.6) | 1.95  p=0.16 |
| NRT Lozenges | 29 (8.1) | 28 (9.0) | 1 (2.2) | 2.49  p=0.14 |
| NRT Inhaler | 4 (1.1) | 3 (1.0) | 1 (2.2) | 0.53  p=0.47 |
| NRT Nasal Spray | 3 (0.8) | 3 (1.0) | 0 (0.0) | 0.47  p=0.50 |
| Prescription Medication - Buproprion | 32 (8.9) | 29 (9.3) | 3 (6.5) | 0.38  p=0.54 |
| Prescription Medication – Varenicline | 29 (8.1) | 27 (8.7) | 2 (4.4) | 1.00  p=0.32 |
| State Quitline | 24 (6.7) | 22 (7.1) | 2 (4.4) | 0.47  p=0.49 |
| Counseling | 31 (8.7) | 28 (9.0) | 3 (6.5) | 0.30  p=0.58 |
| Vape/Electronic cigarettes | 15 (4.2) | 12 (3.8) | 3 (6.5) | 0.71  p=0.40 |
| Cold turkey | 60 (16.8) | 52 (16.7) | 8 (17.3) | 0.02  p=0.90 |
| **Willingness to Use Cessation Methods** |  |  |  |  |
| NRT Patches | 165 (46.1) | 151 (48.4) | 14 (30.4) | 8.79  p=0.01 |
| NRT Gum | 159 (44.4) | 145 (46.5) | 14 (30.4) | 10.04  p=0.01 |
| NRT Lozenges | 150 (41.9) | 138 (44.2) | 12 (26.1) | 10.04  p=0.02 |
| NRT Inhaler | 128 (35.8) | 119 (38.1) | 9 (19.6) | 6.64  p=0.08 |
| NRT Nasal Spray | 84 (23.5) | 79 (25.3) | 5 (10.9) | 9.24  p=0.03 |
| Prescription Medication - Buproprion/Zyban | 112 (31.3) | 103 (33.0) | 9 (19.6) | 5.86  p=0.11 |
| Prescription Medication – Varenicline/Chantix | 103 (28.8) | 94 (30.1) | 9 (19.6) | 5.51  p=0.14 |
| Quitline | 76 (21.2) | 70 (22.4) | 6 (13.0) | 5.28  p=0.15 |
| Counseling | 120 (33.5) | 106 (34.0) | 14 (30.4) | 5.80  p=0.12 |

^a^Categories are not mutually exclusive and participants could report use of more than one type.

n=number of participants; %: percentage; X^2^: chi-square.
